# Periarticular Hyperphosphatemic Familial Tumoral Calcinosis in a Saudi Patient: A Case Report

**DOI:** 10.7759/cureus.84292

**Published:** 2025-05-17

**Authors:** Mahdi Mofarah Alqarni, Sami Amer M Alqarni, Ali Alshareef, Mohammed Omara, Abdullah Asiri, Ali Abdullah Alshehri, Sami Aoudah Aldhabaan

**Affiliations:** 1 Pediatric Orthopedic, Abha Maternity and Children Hospital, Abha, SAU; 2 Orthopedic Surgery, Armed Forces Hospital - Southern Region, Khamis Mushait, SAU; 3 Orthopedic Surgery, Aseer Central Hospital, Abha, SAU

**Keywords:** case report, elbow pain, hip pain, hyperphosphatemic familial tumoral calcinosis, tumoral calcinosis

## Abstract

Hyperphosphatemic familial tumoral calcinosis (HFTC) is a rare autosomal recessive disorder characterized by ectopic calcifications in periarticular soft tissues due to mutations in genes such as GALNT3, FGF23, or KL, leading to FGF23 deficiency or resistance and subsequent hyperphosphatemia. This study describes a 12-year-old girl from Jazan, Saudi Arabia, who presented with progressive right hip pain and swelling, initially managed as an infection. Imaging revealed periarticular calcifications, and laboratory tests confirmed hyperphosphatemia with normal calcium and parathyroid hormone levels. Genetic testing identified a homozygous pathogenic variant in GALNT3, confirming HFTC type 1. Recurrence occurred 1.5 years later in the right elbow, with similar radiographic findings. Further evaluation via CT demonstrated basal ganglia and parotid gland calcifications, highlighting systemic involvement.

Management included complete surgical resection of calcific deposits, followed by acetazolamide (500 mg twice daily) and a low-phosphorus diet. Over one year of multidisciplinary follow-up, no recurrence was observed. Histopathology revealed microcalcifications with a giant cell reaction, consistent with HFTC. HFTC’s diagnosis relies on clinical, biochemical (hyperphosphatemia), and radiological findings (multilobulated periarticular calcifications), supplemented by genetic testing. Treatment involves phosphate-lowering strategies (dietary restriction, phosphate binders, acetazolamide) and surgical excision for symptomatic lesions. This study underscores the importance of early recognition, genetic confirmation, and a multidisciplinary approach to prevent complications and recurrence.

## Introduction

Hyperphosphatemic familial tumoral calcinosis (HFTC) is a rare autosomal recessive disorder distinguished by the presence of ectopic calcifications in periarticular soft tissues, notably surrounding joints like the hips, elbows, and shoulders. The condition occurs due to a relative deficiency or resistance to fibroblast growth factor 23 (FGF23), resulting in hyperphosphatemia [1]. Patients exhibit firm to hard swellings in clinical observations, while laboratory analyses generally indicate elevated phosphate levels alongside normal calcium, vitamin D, and parathyroid hormone levels [2].

The approach to HFTC management includes restricting dietary phosphate, utilizing phosphate binders, and performing surgical removal of calcified lesions when required [2]. Genetic testing is vital in validating the diagnosis, given that gene mutations like GALNT3, Klotho (KL), or FGF23 are frequently linked to HFTC. Recognizing these genetic mutations is crucial for informing long-term management strategies and providing effective genetic counseling [1-3].

Individuals with HFTC demonstrate elevated phosphate levels from enhanced phosphate reabsorption in the proximal tubules while sustaining normal calcium and parathyroid hormone concentrations. Genetic analyses have revealed several mutations linked to HFTC, including a new GALNT3 variant associated with maternal uniparental disomy of chromosome 2 [3].

No established treatment guidelines currently exist. Nonetheless, instances have been documented in which medical interventions have yielded positive outcomes [4-6]. One approach involves reducing intestinal phosphate absorption through dietary restrictions, aiming for a limit of 400 mg per day, supplemented with aluminum hydroxide and/or sevelamer [7]. The administration of acetazolamide is advised for reducing renal phosphate absorption [7,8]. In cases where the lesions result in pain, deformity, or restricted movement, it is advisable to proceed with surgical resection of the deposits [9-13]. Nonetheless, the lesions are likely to reappear if the underlying metabolic imbalance persists [7]. Consequently, combining resection with medical treatment is advisable [14,15].

## Case presentation

The patient is a 12-year-old girl from Jazan, Saudi Arabia, with no significant past medical history. She has a second-degree relative with a history of a benign bone tumor that was resected, although the histopathological diagnosis is unknown.

She came to our hospital after three weeks complaining of right leg pain after being treated elsewhere with amoxicillin/clavulanic acid and a non-steroidal anti-inflammatory drug (NSAID); the pain started gradually and increased in volume over time. During this time, the patient had no fever or constitutional symptoms.

A physical examination found right hip swelling, which demonstrated soft consistency and tenderness; the range of motion was without limitation. There were no reports of infection and no discharge of caseous material. The patient came back after 1.5 years post-resection of the right hip calcinosis, complaining of right elbow pain and with the same clinical picture of the right hip.

**Figure 1:**
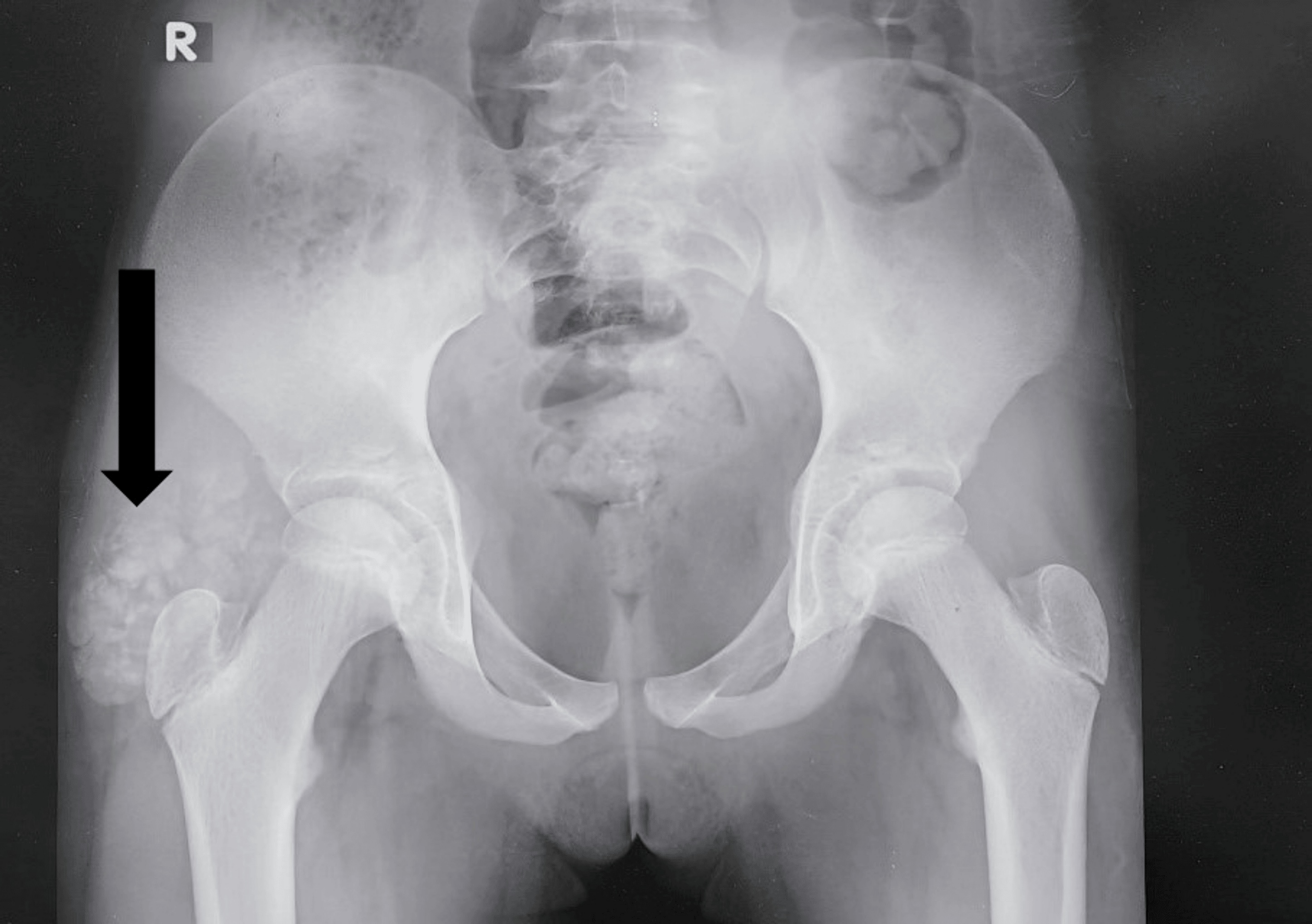
Anteroposterior (AP) X-ray of both hips shows right hip periarticular calcification (arrow).

**Figure 2:**
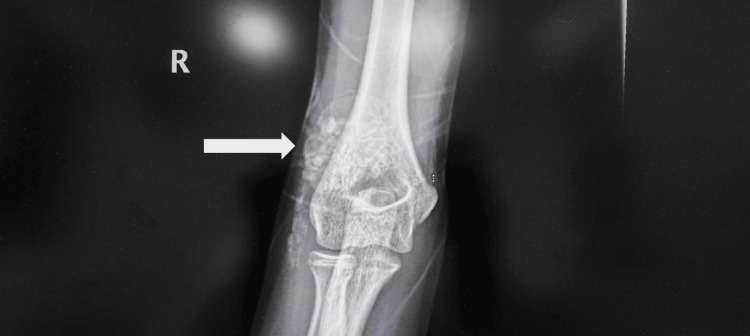
Anteroposterior (AP) X-ray of right elbow shows right elbow periarticular calcification (arrow).

**Figure 3:**
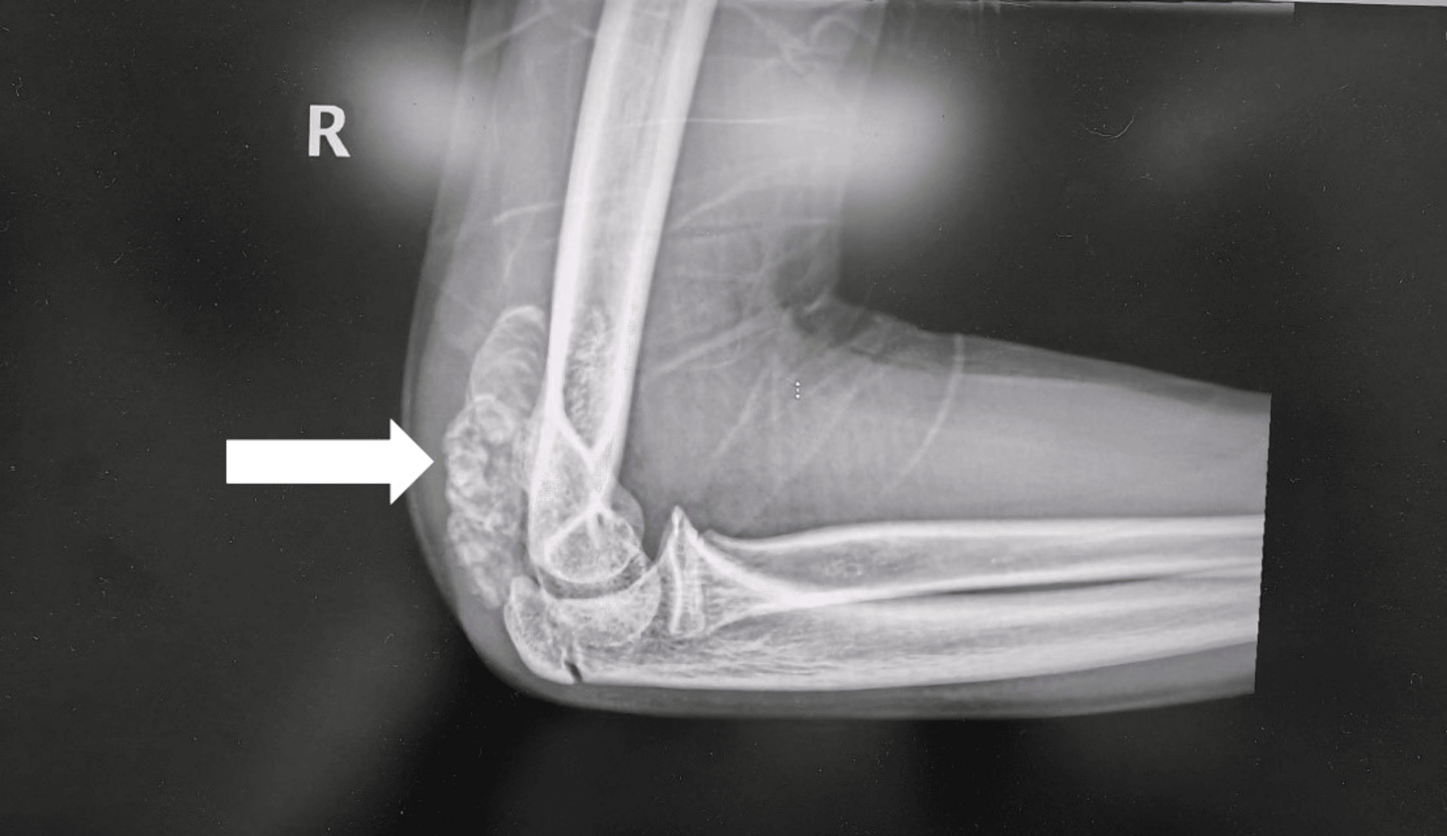
Lateral X-ray of right elbow shows right elbow periarticular calcification (arrow).

X-rays were obtained of the right hip and the right elbow, revealing clusters of numerous, uniform round opacities interspersed with clear radiolucent lines, which appeared independent of and unaffected by the surrounding bone structures (Figures 1-3).

Laboratory results indicated elevated levels of phosphorus and inflammation markers, including C-reactive protein and erythrocyte sedimentation rate (Table 1). A genetic analysis indicated the identification of a homozygous pathogenic variation in the GALNT3 gene. The genetic diagnosis of autosomal recessive hyperphosphatemic familial tumoral calcinosis type 1 has been validated.

**Table 1:** Laboratory results show elevated phosphate levels and increased inflammatory markers. H: high

Test	At the time of right hip complaint	Reference ranges	At the time of right elbow complaint	Reference ranges
WBC	11.98×10^9^/L	4.5-13.5×10^9^/L	8.01×10^9^/L	4.5-13.5×10^9^/L
CRP	47.9 mg/L H	<10 mg/L	5.5 mg/L H	<5 mg/L
ESR	47 mm/h H	<10 mm/h	24 mm/h H	<10 mm/h
Creatinine	45.4 µmol/L	27-88 µmol/L	59.6 µmol/L	27-88 µmol/L
Sodium	139 mmol/L	133-143 mmol/L	139 mmol/L	133-143 mmol/L
Potassium	4.2 mmol/L	3.5-5.1 mmol/L	3.9 mmol/L	3.5-5.1 mmol/L
Calcium (serum)	2.39 mmol/L	2.23-2.58 mmol/L	2.39 mmol/L	2.23-2.58 mmol/L
Phosphate	2.82 mmol/L H	1.72-2 mmol/L	2.5 mmol/L H	1.72-2 mmol/L

**Figure 4:**
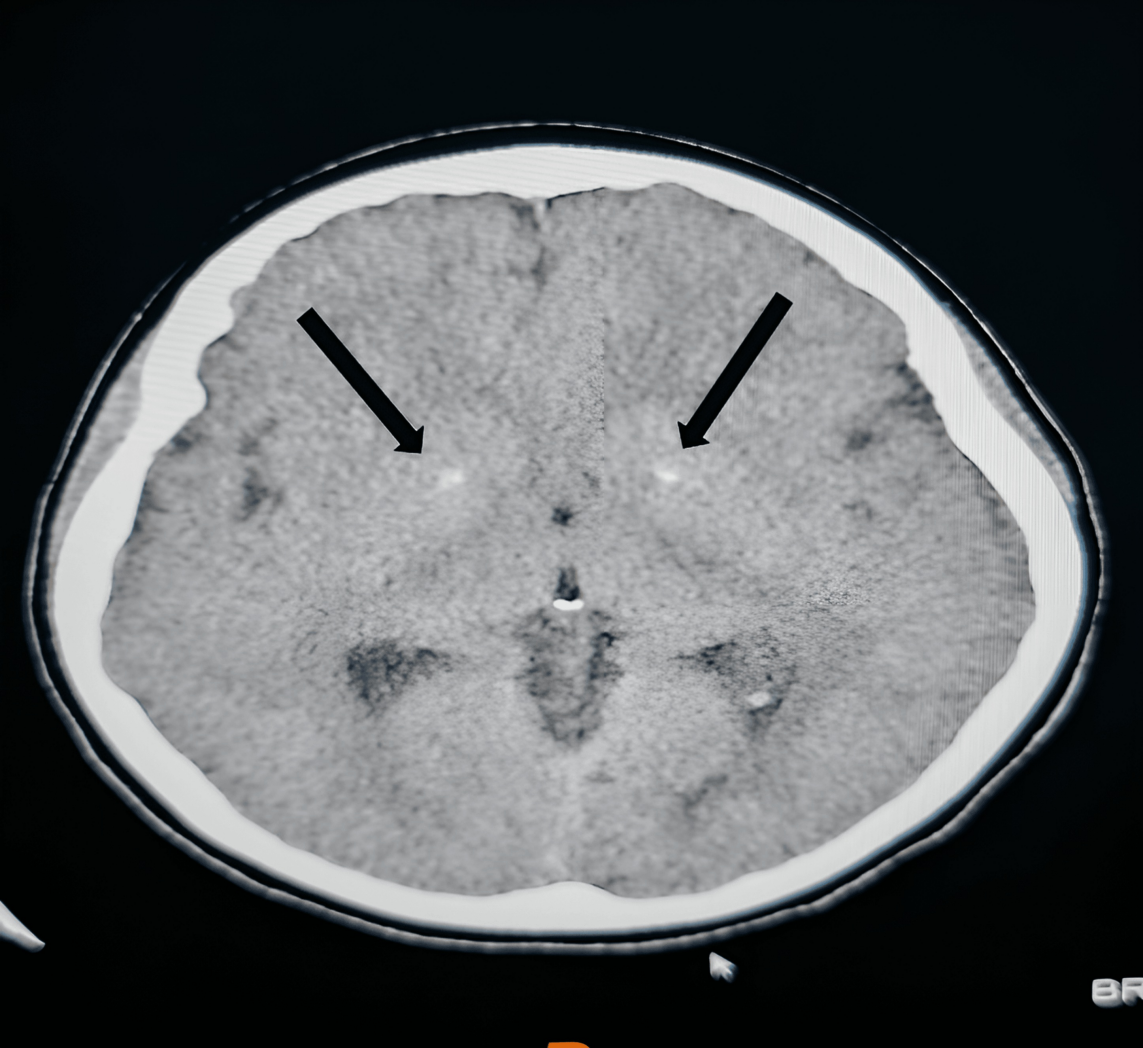
CT brain shows basal ganglia calcification (arrows).

**Figure 5:**
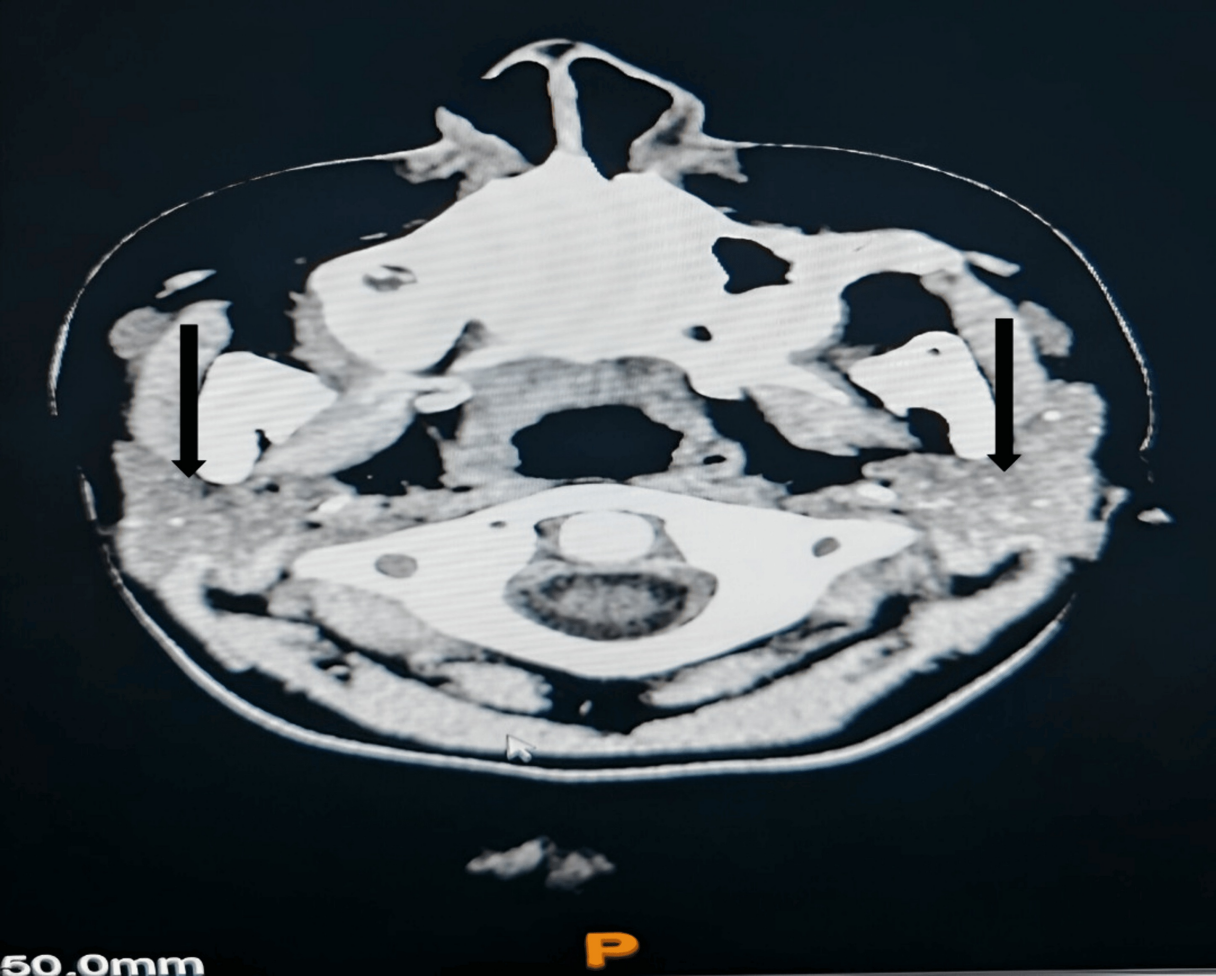
CT brain shows calcification within bilateral parotid gland (arrows).

An echocardiogram and renal ultrasound were performed to evaluate for the presence of calcifications. All studies were reported to be without significant findings. Plain CT of the skull found basal ganglia calcification as well as foci of calcification within the bilateral parotid gland (Figures 4, 5).

**Figure 6:**
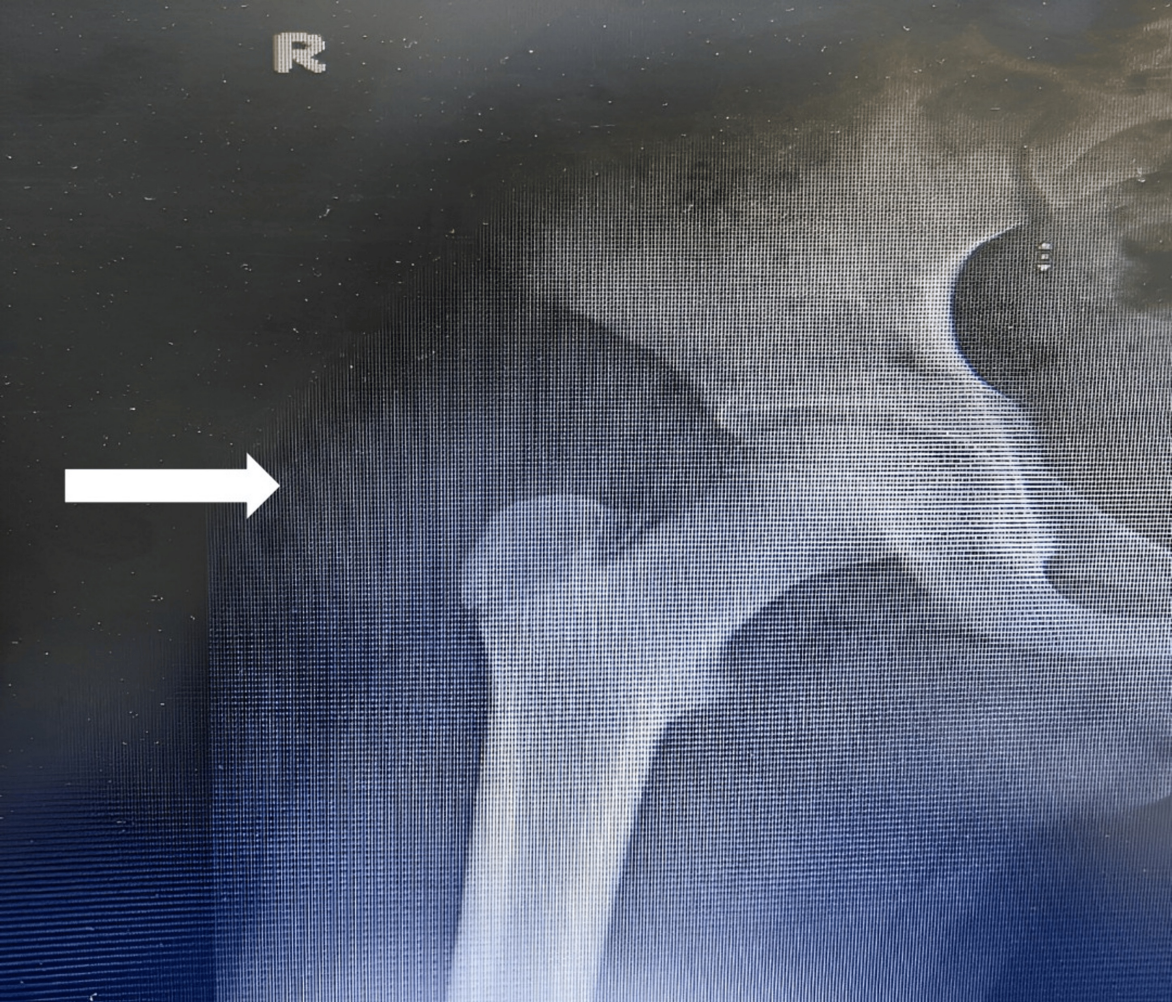
Post-resection AP X-ray right hip shows complete resection (arrow).

**Figure 7:**
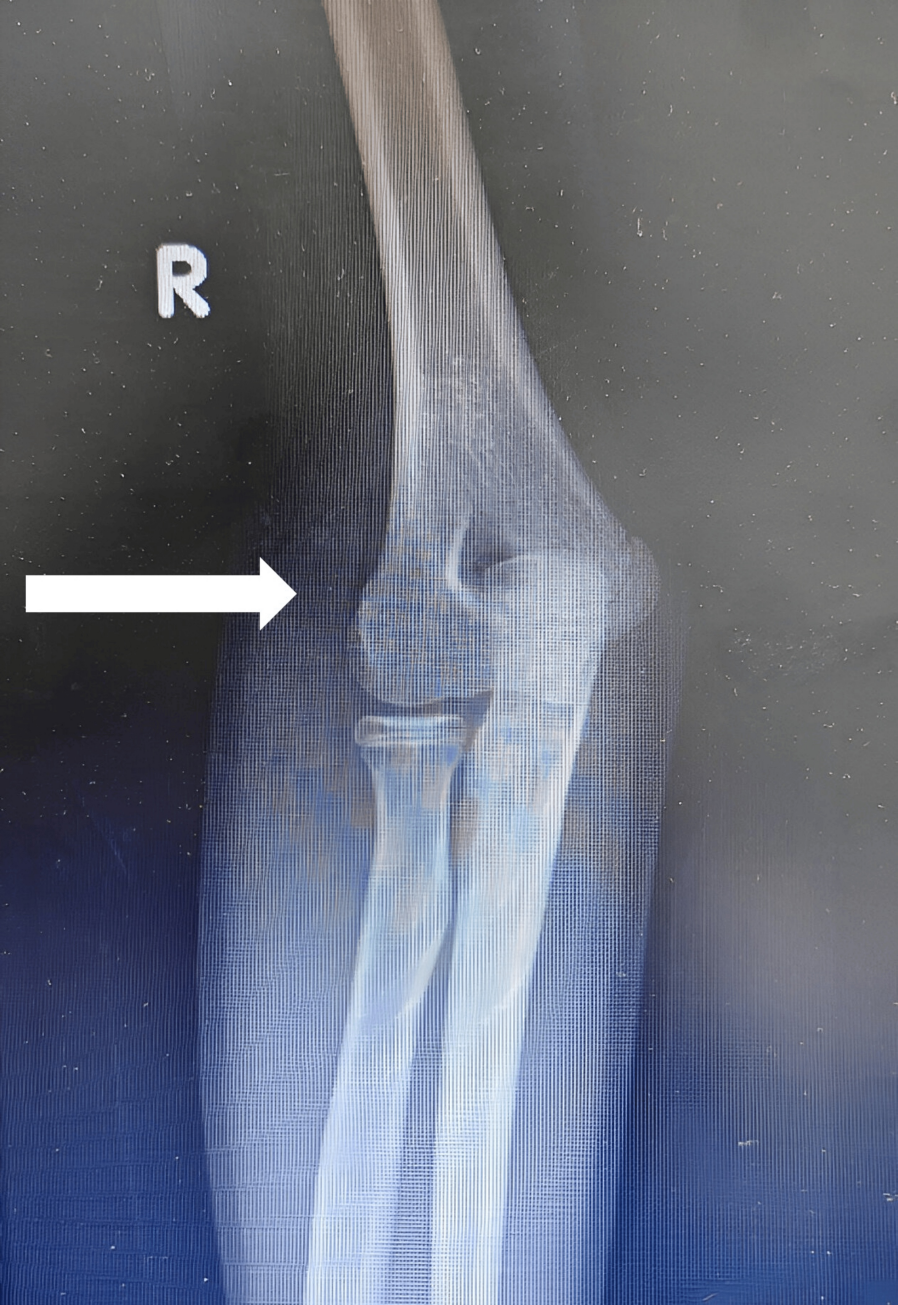
Post-resection AP X-ray right elbow shows complete resection (arrow).

**Figure 8:**
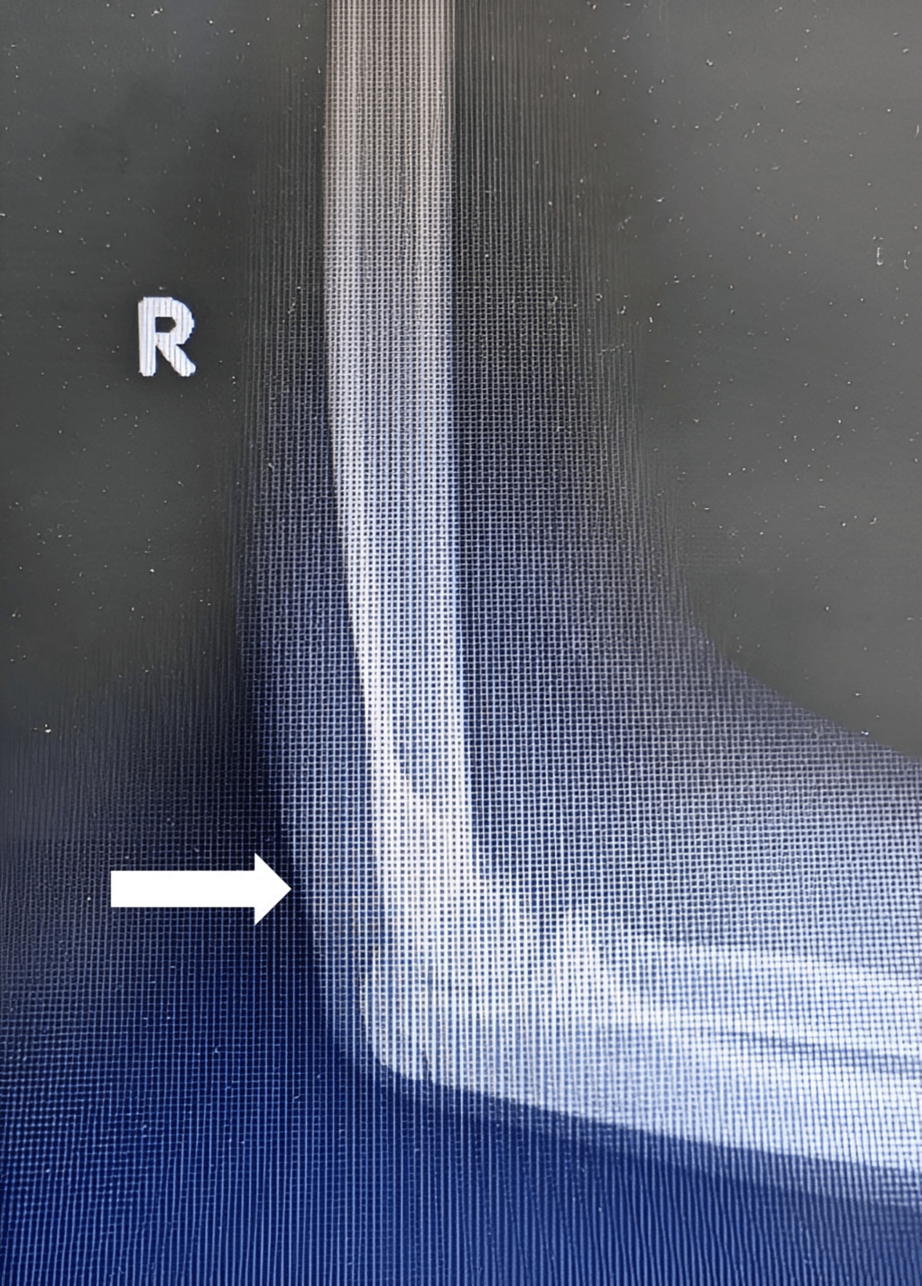
Post-resection lateral X-ray right elbow shows complete resection (arrow).

**Figure 9:**
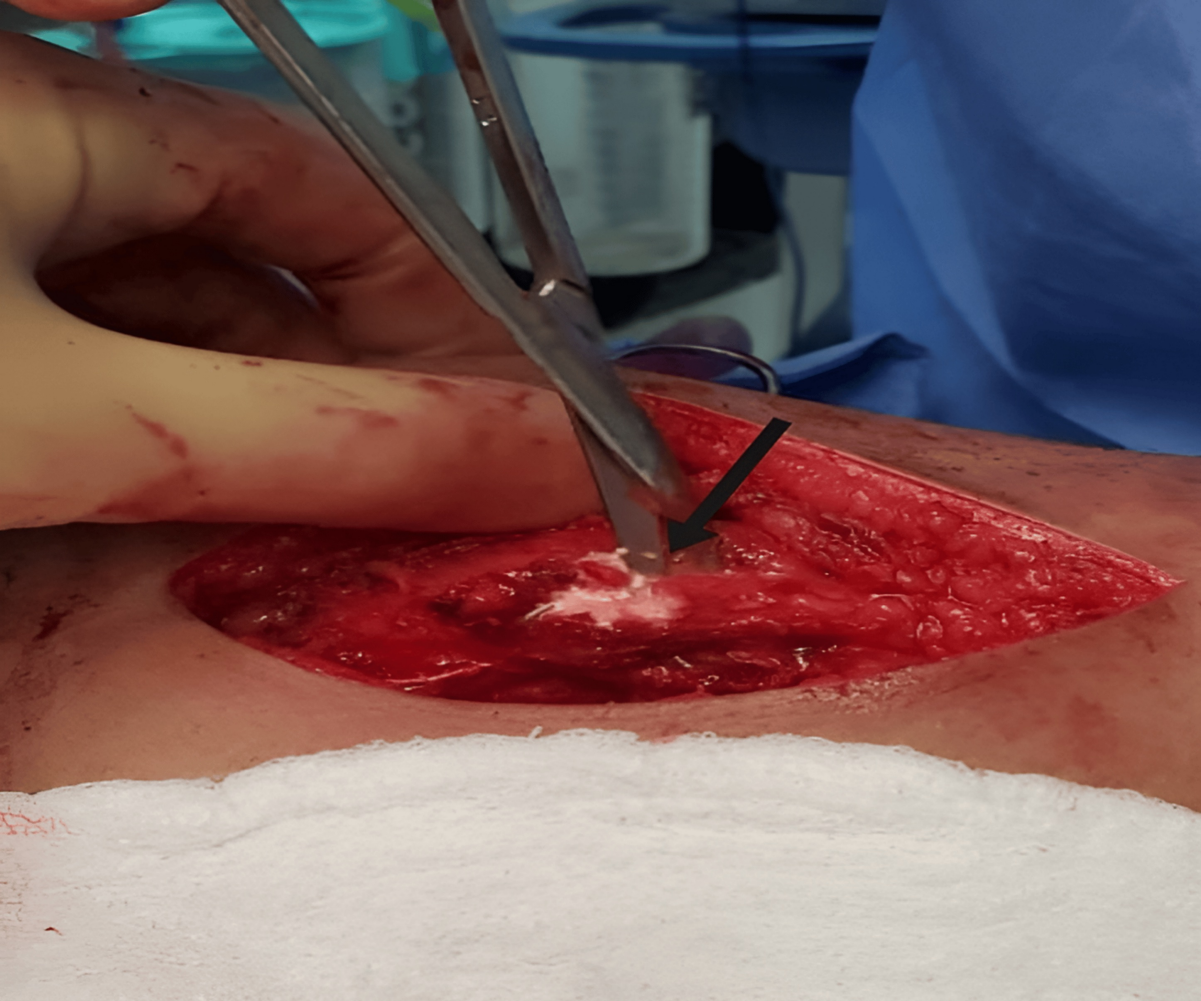
Intra-operative picture of right elbow calcification resection (arrow).

**Figure 10:**
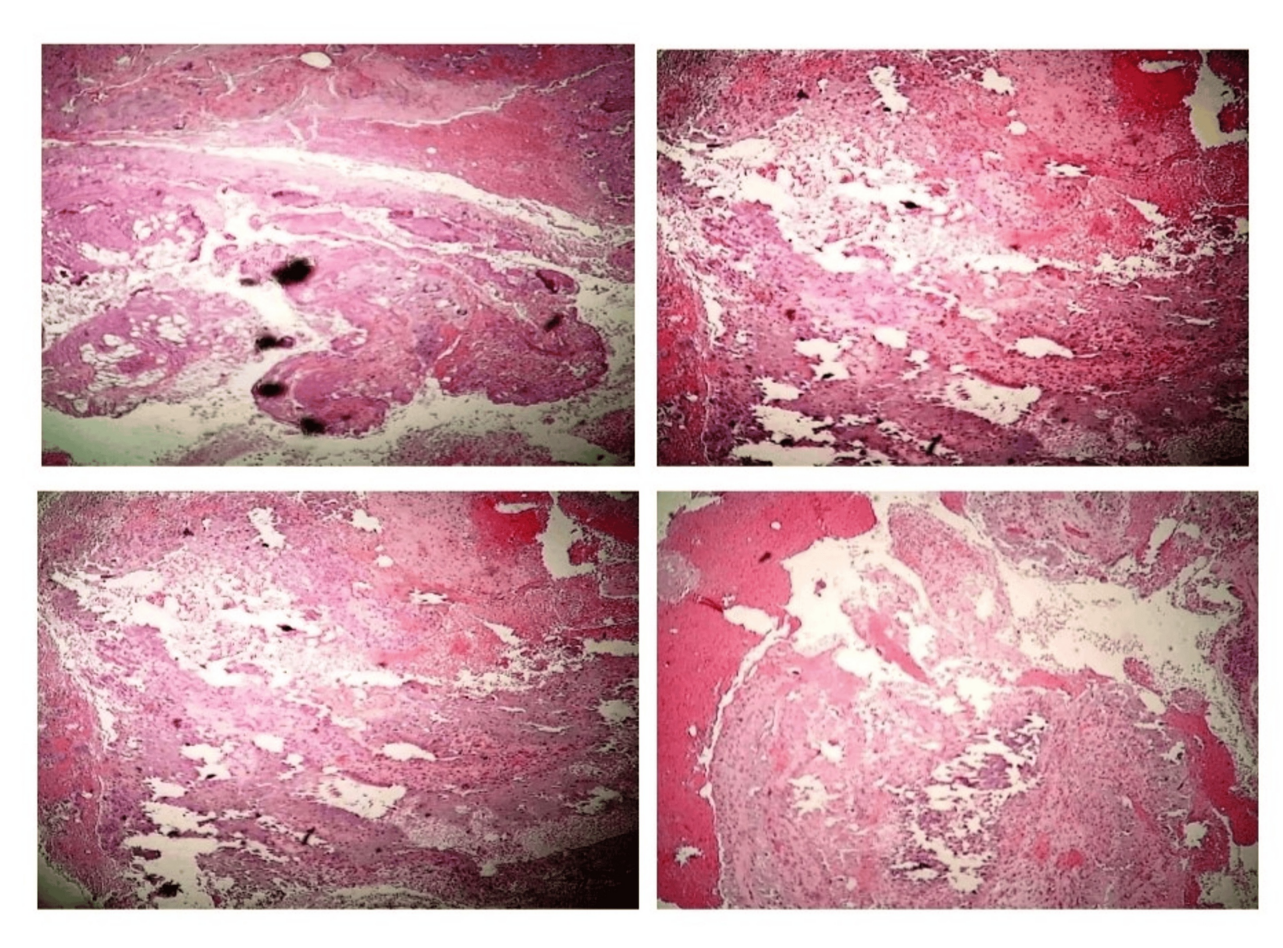
Histopathology slides show microcalcifications with a secondary multinucleated giant cell reaction.

Complete surgical resection of the calcifications in the hip and elbow was performed, and the specimens were sent for pathological examination (Figures 6-8). On macroscopic examination, grayish-white caseous material resembling gout tophi was reported (Figure 9). Histopathological examination showed microcalcifications with secondary multinucleated giant cell reaction (Figure 10).

After the resection of calcifications, the patient was discharged with treatment with 500 mg of acetazolamide twice daily and a diet low in phosphorus. A multidisciplinary team (orthopedic, pediatric, and dietitian) followed up for one year after the beginning of treatment. It was reported that there was no recurrence of the calcifications. The same management was continued.

## Discussion

HFTC is an uncommon syndrome characterized by calcium deposition in soft tissues near the joints. The condition typically begins during adolescence, presenting as painless, firm, tumor-like masses around the joints, which leads to restricted joint function [16]. TC can be classified into normophosphatemic and hyperphosphatemic. Mutations in the genes encoding FGF23, GALNT3, and KL are associated with hyperphosphatemic familial TC. Chronic kidney disease is related to secondary thyroid carcinoma. The characteristic radiographic features of amorphous, cystic, and multilobulated calcifications in a periarticular distribution on extensor surfaces, combined with the biochemical indicator of hyperphosphatemia resulting from enhanced renal phosphate absorption without renal impairment, are diagnostic of HFTC. Genetic analysis may clarify the specific genetic mutation responsible for the metabolic defect. The diagnosis of HFTC is straightforward when the characteristic radiological findings are evident.

In subtle cases, diagnosis may be confirmed using isotope bone scans, CT, or MRI. Histopathological examination reveals multiple cystic spaces with extensive geographic calcification, bordered by palisaded histiocytes and numerous foreign body-type giant cells, serving as confirmatory evidence in ambiguous cases.

Management is multidisciplinary and includes dietary phosphate restriction, the use of phosphate-lowering agents such as aluminum hydroxide and sevelamer, and renal phosphate excretion promoters like acetazolamide, which induces phosphaturia. This combined approach may have a beneficial synergistic effect in reducing hyperphosphatemia and preventing recurrence. Surgical excision is warranted in large lesions that restrict movement or are accompanied by an infection [16].

## Conclusions

Because of its non-specific presentation and similarity with common musculoskeletal disorders, hyperphosphatemic familial tumoral calcinosis (HFTC) warrants heightened clinical suspicion. To confirm hyperphosphatemia and ectopic calcifications, a prompt and accurate diagnosis requires a combination of genetic analysis, radiographic imaging, and biochemical testing. Due to the systemic complexity of this rare autosomal recessive disorder, management necessitates a multidisciplinary team approach tailored to the patient's clinical presentation. This team typically includes a dietitian, pediatrician, endocrinologist, cardiologist, orthopedic surgeon, nephrologist, and geneticist.

Optimizing phosphate, slowing the progression of ectopic calcification, offering genetic counseling, and putting in place long-term surveillance for complications are important management priorities. Ultimately, this collaborative framework enhances outcomes for affected individuals and their families by enabling personalized patient care and advancing our understanding of the pathophysiology of HFTC.
